# Optical Properties of New Third-Order Nonlinear Materials Modified by Click Chemistry

**DOI:** 10.3390/molecules27155006

**Published:** 2022-08-06

**Authors:** Yuzhen Zhao, Zhenhua Li, Qing Li, Yang Zhao, Ruijuan Yao, Cheng Ma, Yongming Zhang, Dong Wang

**Affiliations:** 1 Xi’an Key Laboratory of Advanced Photo-Electronics Materials and Energy Conversion Device, School of Electronic Information, Xijing University, Xi’an 710123, China; 2 Department of Materials Physics and Chemistry, School of Materials Science and Engineering, University of Science and Technology Beijing, Beijing 100083, China

**Keywords:** third-order nonlinear, click chemistry, optical properties

## Abstract

A high-yielding click reaction was used to synthesize a series of highly conjugated, symmetrical, as well as asymmetrical compounds with a benzene core. Cyclic voltammetry and ultraviolet/visible absorption spectroscopy were carried out, and proved that the side groups of the benzene derivatives played an important role in the energy gaps, and affected the third-order non-linear optical response. The maximum absorption wavelength of the series of benzene derivatives showed an obvious red-shift. Moreover, the addition of resilient electron-withdrawing groups significantly narrowed the energy levels as compared with precursors. The third-order nonlinear properties of this benzene derivative were tested by the Z-scan technique. The expected properties of this series of molecules were obtained, and it was found that the series of molecules undergoes a transition from reverse saturable absorption to saturable absorption, which has certain reference significance for a nonlinear optical field.

## 1. Introduction

Currently, organic materials are being widely studied for their third-order nonlinear optical (NLO) properties thanks to the great nonlinear figure, high damage verge, super quick response time, and ability to modify material properties (molecular engineering) [1,2]. For photonic applications, resources with enormous third-order ocular nonlinearities, as well as debauched nonlinear optical properties, are found to be very important. Photonic applications such as statistics dispensation, ocular substituting, and plus communication are closely related to the NLO phenomenon [3,4,5,6]. It is of great significance to prepare suitable nonlinear optical materials for the future development of photonic applications. Besides, organic materials have great potential in nonlinear optical materials [7,8]. Based on this, there is urgency among scientists to find organic materials with a high non-linear response. To obtain detailed information about the properties of these molecules, structural changes are made in building blocks of donor and acceptors plus π-units (conjugated linking) [9,10,11].

The high conjugated π-system and long aromatic core in the molecular structure of organic molecules complemented the large NLO properties [12,13]. As a matter of fact, there are many organic molecules that have been studied in the field of nonlinear optics, such as porphyrin derivatives [14] and fullerene derivatives [15,16] with high conjugated structures. Comparing large conjugated molecules with the core of porphyrin and fullerene, highly conjugated molecules with the core of benzene can be synthesized and purified easily.

Click reaction is important because of its high selectivity, effectiveness, and lack of end-products [17]. [2 + 2] click reactions represent a one-step and great yielding approach. Products of this reaction are generally stable and decontaminated very easily after the introduction of click reagents. The most convenient method for the preparation of chromophores reported so far is ceremonial [2 + 2] CA–CR (cycloaddition–cycloreversion) responses. This reaction occurs between alkynes and electron-deficient alkenes, including tetracyanoethene (TCNE), tetracyanoquiodimethane (TCNQ), and 2,3,5,6-tetrafluoro-7,7,8,8-tetracyanoquinodime-thane (F4-TCNQ) [18,19,20,21]. Chromophores were non-polar, π-conjugated, and donor–acceptor. They have truncated vigor and intramolecular CT (charge transfer), and thus changed the conjugated structure of the molecule, which further affected the third-order NLO properties of the material [22,23]. Therefore, click reaction has great potential to be an admirable channel to develop a post-functionalization method that might design and synthesize new third-order NLO materials by electron-rich alkynes reacting with strong acceptor molecules [24].

The difficulty in the synthesis and purification of NLO materials limits its wide range of applications. In this paper, simple and highly efficient nonlinear materials were reported to solve this problem. Based on the sequence and sum of click moieties, various properties like electron-withdrawal and various conjugated π-systems of new compounds were discussed. Future research should employ these novel compounds in many other electronic fields in the future to make our lives smarter.

## 2. Experimental

All reagents were bought from Aladdin (commercial site). No further purification processes were carried out. Reagents were directly used in experiments. Bruker Avance III HD NMR 500 MHZ at 20 °C, Perkin Elmer LR-64912C, JASCO V-570, and Shimadzu AXIMA-CFR spectrometers were used to measure 1H NMR, FT-IR, UV/Vis, and MALDI-TOF-MS continuums, respectively. The internal reference of the solvent’s enduring signals and SiMe_4_ for downfield was used to measure chemical shifts (ppm).

This experiment was performed at 20 kV potential with direct +ve ion mode. A dithranol matrix was used. Flash EA 1112 instrument was used for the elemental study. A conventional cell with three electrodes was used for measuring cyclic voltammetry. The working electron was glass carbon (2 mm) and the counter electrode was platinum, while the reference electrode was silver/silver chloride. The experiment was conducted on a CHI 660C instrument (computer-controlled). Fc/Fc^+^ (ferrocenium/ferrocene) was used as an internal standard. All of the potentials used were referenced to it. The Z-scan method (20 ps/532 nm) was used for studying the response of the NLO properties with a mode-locked Nd/YAG laser.

### 2.1. 4,4′-(1,4-Phenylenebis(ethyne-2,1-diyl))bis(N,N-dihexadecylaniline) (***B***)

To prepare 4,4′-(1,4-phenylene bis(ethyne-2,1-diyl))bis(N,N-dihexadecylaniline) (**B-23**), 0.10 (0.79 mmol) and 1.06 g (1.58 mmol) of 1,4-diethynylbenzene and N,N-dihexadecyl-4-iodoaniline), respectively, were dissolved in 8 milliliters of 1:1 mixed solution of tetrahydrofuran (THF) and triethylamine (TEA). Then, this mixture was exposed to argon for half an hour following the addition of Pd (PPh_3_)_2_ @ 77.0 milligrams (0.07 millimole) and copper iodide (26.6 milligrams, 0.14 mmole).

It was then shaken for 10 h at 40 °C under argon. The mixture was then resolute, following the dilution with CH_2_Cl_2_. Plugs of silica gel were used for filtration. Flush was detached in vacuo. Product purification was carried out using column chromatography ((SiO_2_, petroleum ether/dichloromethane = 8:1)). Yellow powder was obtained as an end-product of this reaction. This yellow powder is **B-23** (714.0 mg, 75%). ^1^H NMR (deuterated chloroform, 500 MHz): δ = 0.880 (12 h, t, J = 7.0 Hz), 1.30 (104H, m), 1.57 (8H, m), 3.40 (8H, m), 6.60 (4H, d, J = 8.5 Hz), 7.36 (4H, d, J = 8.0 Hz), 7.42 (4H, s) parts per million. Fourier transformed infra-red (potassium bromide/cm): v = 2930, 2848, 2210, 1609, 1527, 1471, 1372, 1200, 1152, 824, 724. MALDI-TOF-MS (dithranol): m/z: calculated C_86_H_144_N_2_: 1205.13 g/mole, obtained: 1205.2 g/mole [M]. C_86_H_144_N_2_ (1205.13). Fundamental examination (%): carbon = 85.64, H = 12.03, and nitrogen = 2.23; obtained: carbon = 85.64, hydrogen = 12.05, and nitrogen = 2.24.

### 2.2. 2-(4-(Dihexadecylamino)phenyl)-3-(4-((4(dihexadecylamino)phenyl)ethynyl)phenyl)buta-1,3-diene-1,1,4,4-tetracarbonitrile (***B-1***)

To prepare (**B-1**), 150.0 mg of a click monomer **B-23** solution with a molar concentration of 0.12 millimole in 8 mL of CH_2_Cl_2_ was taken. Then, 15.36 mg of TCNE (0.12 mmol) (click reagent) was added. This mixture was shaken for one hour in the air. Flush vanished while the filtrate remained, which was then decontaminated using column chromatography (SiO_2_, CH_2_Cl_2_). The yellow-brown solid product was **B-1** (148.8 mg, 90%) ^1^H NMR (Deuterated chloroform, 500 MHz): δ = 0.88 (12H, t, J = 7.0 Hz), 1.30 (104H, m), 1.57 (8H, m), 3.30 (4H, t, J = 5.0 Hz), 3.40 (4H, m), 6.58 (2H, d, J = 8.5 Hz), 6.67 (2H, d, J = 10.0 Hz), 7.38 (2H, d, J = 8.5 Hz), 7.57 (2H, d, J = 8.0 Hz), 7.73 (2H, d, J = 8.0 Hz), 7.78 (2H, d, J = 9.0 Hz) parts per million. Fourier transformed infra-red (potassium bromide/cm): v = 2930, 2848, 2208, 1600, 1490, 1353, 1190, 1152, 816, 723. MALDI-TOF-MS: calculated C_92_H_144_N_6_: 1334.15 g/mol, obtained: 1334.8 g/mole [MH]^+^; basic investigation (%) of C_92_H_144_N_6_ (1334.15): carbon = 82.82, hydrogen = 10.88, and nitrogen = 6.30; obtained: carbon = 82.83, hydrogen = 10.89, and nitrogen = 6.41.

### 2.3. 3′-(1,4-Phenylene)bis(2-(4-(dihexadecylamino)phenyl)buta-1,3-diene-1,1,4,4-tetracarbonitrile) (***B-11***)

All of the experimental conditions were the same as **B-1**, except for the molar concentration of click reagent, which was twice that of **B-1**.

After solvent removal, brown solid was obtained by purifying the raw product using column chromatography.

This brown solid is **B-11** (169.9 mg, 94%). ^1^H NMR (deuterated chloroform, 500 MHz): δ = 0.88 (12H, t, J = 7.0 Hz), 1.30 (104H, m), 1.57 (8H, m), 3.40 (8H, m), 6.71 (4H, d, J = 9.0 Hz), 7.79 (4H, d, J = 8.5 Hz), δ = 7.81 (4H, s) parts per million (ppm). Fourier transformed infra-red (potassium bromide/cm): v = 2930, 2848, 2217, 1600, 1490, 1334, 1190, 1152, 824, 722. MALDI-TOF-MS: calculated (g/mol) C_98_H_144_N_10_: 1462.16, obtained: 1462.2 [M]; elemental analysis (%) of C_98_H_144_N_10_ (1462.16): carbon = 80.50, hydrogen = 9.93, and nitrogen = 9.58; obtained: carbon = 80.40, hydrogen = 10.01, and nitrogen = 9.61.

### 2.4. 2-(4-(3,3-Dicyano-2-(4-(dihexadecylamino)phenyl)-1-(4-((4-(dihexadecylamino)phenyl)ethynyl)phenyl)allylidene)cyclohexa-2,5-dien-1-ylidene)malononitrile (***B-2***)

To synthesize **B-2**, 16.40 mg of reagent (TCNQ, 0.08 millimole) was dissolved in the amalgam of **B-23**. It contained 100.0 mg of **B-23** (click monomer) dissolved in 8 mL of dichloromethane. It was then agitated for one hour at 30 °C in air. The solvent was separated while the final product was obtained after purification using column chromatography (SiO_2_, dichloromethane). Black green solid (101.6 mg, 90%) was obtained, which is called **B-2**.

^1^H NMR (deuterated chloroform, 500 MHz): δ = 0.88 (12H, t, J = 7.0 Hz), 1.30 (104H, m), 1.57 (8H, m), 3.31 (4H, m), 3.39 (4H, m), 6.59 (2H, d, J = 8.0 Hz), 6.68 (2H, d, J = 8.5 Hz), 6.98 (1H, d, J = 10.0 Hz), 7.18 (1H, d, J = 9.0 Hz), 7.26 (2H, m), 7.38 (2H, d, J = 8.5 Hz), 7.52 (4H, m), 7.67 (2H, d, J = 8.5 Hz) parts per million (ppm). Fourier transformed infra-red (potassium bromide/cm): v = 2930, 2847, 2209, 1581, 1518, 1408, 1353, 1180, 824. MALDI-TOF-MS: calculated (g/mol) C_98_H_148_N_6_: 1410.18, obtained: 1410.1 [M]; elemental analysis (%) of C_98_H_148_N_6_ (1410.18): carbon = 83.46, hydrogen = 10.58, and nitrogen = 5.96; obtained: carbon = 83.41, hydrogen = 10.54, and nitrogen = 5.97.

### 2.5. 2-(4-(3,3-Dicyano-1-(4-(1,1-dicyano-3-(4-(dicyanomethylene)cyclohexa-2,5-dien-1-ylidene)-3-(4-(dihexadecylamino)phenyl)prop-1-en-2-yl)phenyl)-2-(4-(dihexadecylamino)phenyl)allylidene)cyclohexa-2,5-dien-1-ylidene)malononitrile (***B-22***)

All of the experimental conditions were the same as **B-2**, except for the molar concentration of click reagent, which was twice that of **B-2**.

After solvent removal, a light blue solid was obtained by purifying the raw product using column chromatography. This light blue solid is **B-22** (122.2 mg, 92%).

^1^H NMR (deuterated chloroform, 500 MHz): δ = 0.88 (12H, t, J = 7.0 Hz), 1.30 (104H, m), 1.57 (8H, m), 3.39 (8H, m), 6.70 (4H, d, J = 8.5 Hz), 6.95 (2H, d, J = 8.5 Hz), 7.15 (2H, d, J = 8.5 Hz), 7.24 (4H, d, J = 8.5 Hz), 7.49 (4H, m), 7.76 (4H, s) parts per million (ppm). Fourier transformed infra-red (potassium bromide/cm): v = 2930, 2848, 2208, 1590,1515, 1405, 1350, 1180, 823. MALDI-TOF-MS: calculated (g/mol), for C_110_H_152_N_10_: 1614.22, obtained: 1614.1 g mol^−1^ [M]; elemental analysis (%) of C_110_H_152_N_10_ (1614.22): carbon = 81.83, hydrogen = 9.49, and nitrogen = 8.68; found: carbon = 81.82, hydrogen = 9.50, and nitrogen = 8.67.

### 2.6. 2-(4-(3,3-Dicyano-2-(4-(dihexadecylamino)phenyl)-1-(4-((4-(dihexadecylamino)phenyl)ethynyl)phenyl)allylidene)-2,3,5,6-tetrafluorocyclohexa-2,5-dien-1-ylidene)malononitrile (***B-3***)

To prepare **B-3**, all of the experimental conditions were the same as **B-2**, except for the click reagent. 4F-TCNQ was used as a click reagent in this reaction instead of TCNQ. After solvent removal, a light blue solid was obtained by purifying the raw product using column chromatography (SiO_2_, C_4_H_8_O_2_). This dark brown solid is **B-3** (109.9 mg, 90%). ^1^H NMR (Deuterated chloroform, 500 MHz): δ = 0.88 (12H, t, J = 7.0 Hz), 1.30 (104H, m), 1.57 (8H, m), 3.30 (4H, m), 3.55 (4H, m), 6.60 (2H, d, J = 8.5 Hz), 6.82 (2H, d, J = 8.5 Hz), 7.38 (4H, d, J = 8.5 Hz), 7.56 (4H, s) parts per million (ppm). Fourier transformed infra-red (potassium bromide/cm): v = 2930, 2847, 2209, 1600, 1389, 1353, 1189, 823, 724. MALDI-TOF-MS: calculated (g/mol) C_98_H_148_F_4_N_6_: 1482.14, obtained: 1482.2 [M]; elemental analysis (%) of C_98_H_148_F_4_N_6_ (1482.14): carbon = 79.41, H = 9.79, and nitrogen = 5.67; obtained: carbon = 79.31, hydrogen = 9.80, and nitrogen = 5.66.

### 2.7. 2-(4-(3,3-Dicyano-1-(4-(1,1-dicyano-3-(4-(dicyanomethylene)-2,3,5,6-tetrafluorocyclohexa-2,5-dien-1-ylidene)-3-(4-(dihexadecylamino)phenyl)prop-1-en-2-yl)phenyl)-2-(4-(dihexadecylamino)phenyl)allylidene)-2,3,5,6-tetrafluorocyclohexa-2,5-dien-1-ylidene)malononitrile (***B-33***)

**B-33** was synthesized similarly to **B-3**. The only difference between the two was the molar concentration of click reagent, which was twice that of **B-3**.

After solvent removal, a purple solid was obtained by purifying the raw product using column chromatography SiO_2_, C_4_H_8_O_2_/ dichloromethane = 1:1.

This purple solid is **B-33** (119.8 mg, 90%). ^1^H NMR (deuterated chloroform, 500 MHz): δ = 0.88 (12H, t, J = 7.0 Hz), 1.30 (104H, m), 1.57 (8H, m), 3.59 (8H, m), 6.85 (4H, m), 7.28 (4H, m), 7.72 (4H, m) parts per million (ppm). Fourier transformed infra-red (potassium bromide/cm): v = 2930, 2847, 2209, 1600, 1389, 1353, 1189, 823, 725. MALDI-TOF-MS: calculated (g/mol) C_110_H_144_F_8_N_10_: 1758.15, obtained: 1759.0 [MH]^+^; elemental analysis (%) of C_110_H_144_F_8_N_10_ (1758.15): carbon = 75.14, hydrogen = 8.25, and nitrogen = 7.97; obtained: carbon = 75.12, hydrogen = 8.23, and nitrogen = 7.94.

### 2.8. 2-(4-(3,3-Dicyano-1-(4-(dicyanomethylene)cyclohexa-2,5-dien-1-ylidene)-2-(4-(dihexadecylamino)phenyl)allyl)phenyl)-3-(4-(dihexadecylamino)phenyl)buta-1,3-diene-1,1,4,4-tetracarbonitrile (***B-12***)

To prepare **B-12**, the same experimental conditions were followed as used for **B-2**, except that **B-1** was used as a click monomer.

Flush vanished while filtrate remained, which was then decontaminated using column chromatography (silica, dichloromethane 1:4) to obtain **B-12**, which was a green solid (114.0 mg, 90%). ^1^H NMR (deuterated chloroform, 500 MHz): δ = 0.88 (12H, t, J = 7.0 HZ), 1.30 (104H, m), 1.57 (8H, m), 3.41 (8H, m), 6.71 (4H, m), 6.90 (1H, d, J = 6.0 Hz), 7.20 (1H, d, J = 9.5 Hz), 7.52 (2H, m), 7.80 (8H, m) parts per million (ppm). Fourier transformed infra-red (potassium bromide/cm): v = 2930, 2847, 2209, 1574, 1410, 1340, 1187, 824, 723. MALDI-TOF-MS: calculated C_104_H_148_N_10_: 1538.19 g/mol, obtained: 1538.2 g/mol [M]; elemental analysis (%) of C_104_H_148_N_10_ (1538.19): carbon = 81.20, hydrogen = 9.70, and nitrogen = 9.10; obtained: carbon = 81.10, hydrogen = 9.68, and nitrogen = 9.12.

### 2.9. 2-(4-(3,3-Dicyano-1-(4-(dicyanomethylene)-2,3,5,6-tetrafluorocyclohexa-2,5-dien-1-ylidene)-2-(4-(dihexadecylamino)phenyl)allyl)phenyl)-3-(4-(dihexadecylamino)phenyl)buta-1,3-diene-1,1,4,4-tetracarbonitrile (***B-13***)

All of the experimental conditions were the same as **B-3**, except that **B-1** was used as a click monomer. Flush vanished while filtrate remained, which was then decontaminated using column chromatography (SiO_2_, dichloromethane) to obtain **B-13** as an amaranthine solid (120.5 mg, 91%). ^1^H NMR (deuterated chloroform, 500 MHz): δ = 0.88 (12H, t, J = 7.0 Hz), 1.30 (104H, m), 1.57 (8H, m), 3.44 (4H, m), 3.57 (4H, m), 6.75 (2H, d, J = 9.0 HZ), 6.82 (2H, d, J = 9.0 Hz), 7.75 (2H, d, J = 8.0 Hz), 7.82 (6H, m) parts per million (ppm). Fourier transformed infra-red (potassium bromide/cm): v = 2930, 2847, 2209, 1599, 1386, 1353, 1187, 1023. MALDI-TOF-MS: calculated C_104_H_144_F_4_N_10_: 1610.15 g/mol, recorded: 1610.1 g/mol [M]; elemental analysis (%) of C_104_H_144_F_4_N_10_ (1610.15): carbon = 77.57, hydrogen = 9.01, and nitrogen = 8.70; found: carbon = 77.60, hydrogen = 9.03, and nitrogen = 8.69.

### 2.10. (E)-2-(4-(3,3-Dicyano-1-(4-(3,3-dicyano-1-(4-(dicyanomethylene)-2,3,5,6-tetrafluorocyclohexa-2,5-dien-1-ylidene)-2-(4-(dihexadecylamino)phenyl)allyl)phenyl)-2-(4-(dihexadecylamino)phenyl)allylidene)cyclohexa-2,5-dien-1-ylidene)malononitrile (***B-23***)

For synthesizing **B-23**, all of the experimental conditions were kept the same as used earlier for **B-3**. The only difference between the two was the click monomer. Instead of **B-23**, **B-2** was used as a click monomer in this reaction. After purification, amaranthine solid (124.7 mg, 90%) was obtained, which is **B-23**. Product purification was carried out similarly as described in previous sections.

^1^H NMR (deuterated chloroform, 500 MHz): δ = 0.88 (12H, t, J = 7.0 Hz), 1.30 (104H, m), 1.57 (8H, m), 3.41 (4H, m), 3.58 (4H, m), 6.73 (2H, d, J = 8.5 Hz), 6.84 (2H, d, J = 8.5 Hz), 6.90 (1H, d, J = 10.0 Hz), 7.16 (1H, d, J = 8.0 Hz), 7.28 (5H, m), 7.70 (1H, d, J = 8.5 Hz), 7.71 (4H, d, J = 8.5 Hz), 7.80 (4H, d, J = 8.5 Hz) parts per million (ppm). Fourier transformed infra-red (potassium bromide/cm): v = 2930, 2847, 2209, 1597, 1388, 1350, 1181, 824, 723. MALDI-TOF-MS: calculated C_110_H_148_F_4_N_10_: 1686.19 g/mol, obtained: 1686.2 g/mol [M]; elemental analysis (%) of C_110_H_148_F_4_N_10_ (1686.19): carbon = 78.34, hydrogen = 8.85, and nitrogen = 8.31; found: carbon = 78.32, hydrogen = 8.83, and nitrogen = 8.32.

## 3. Result and Discussion

### 3.1. Preparation Method

**Scheme 1:**
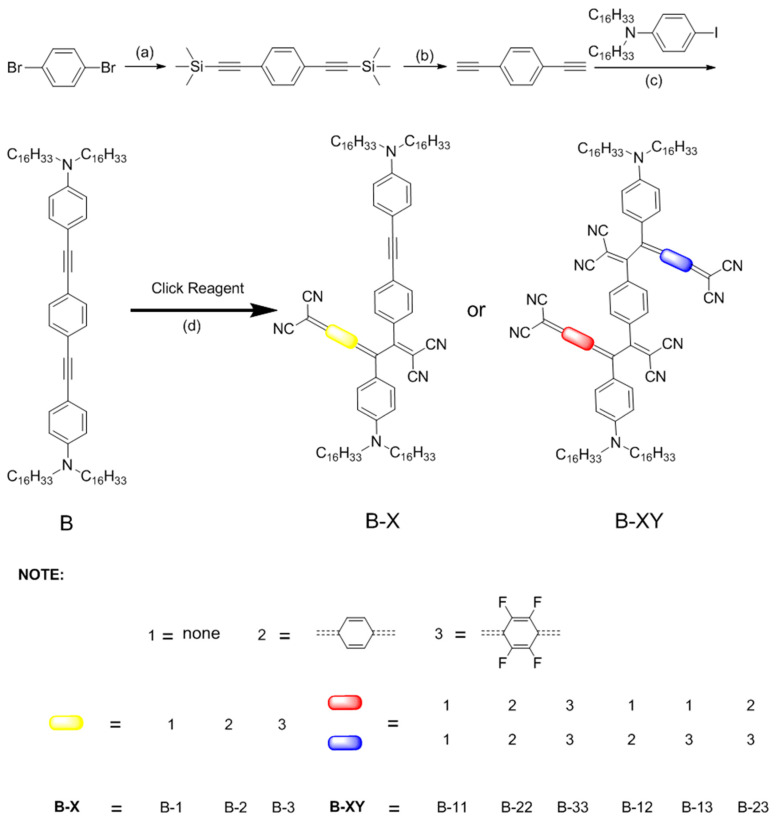
Synthesis of B to B-X and B-XY. (**a**) TMSA, PdCl_2_(PPh_3_)_2_, CuI, tetrahydrofuran (THF), triethylamine (TEA), 80 °C, Ar, 720 min; (**b**) potassium carbonate, methanol, Oxolane, 25 °C, 120 min; (**c**) PdCl_2_(PPh_3_)_2_, CuI, TEA, Oxolane, 40 °C, Ar, 10 h; (**d**) click reagent (TCNE, TCNQ, 4F-TCNQ), dichloromethane (CH_2_Cl_2_), rt, 30 min.

The overall synthetic scheme is depicted in Scheme 1. The highlighted portion shows the transfer of charges between benzene complexes [25]. Cross-coupling (sequential Sonogashira) [26,27,28], as well as silyl deprotection [29,30], were used for the synthesis of compound **B-23**. One B molecule had two acetylene groups that could react with click reagents.

[2 + 2] click reactions (high yielding) were used for the synthesis of compound B-X using one equivalent 4F-TCNQ, TCNQ, and TCNE. They reacted with the acetylene groups to yield **B-1**, **2**, and **3**. B-XY was synthesized through two acetylene groups reacting with the same or different click reagents (TCNE, TCNQ, or 4F-TCNQ), e.g., the symmetrical compound **B-11** was the product of B reacting with two equivalent TCNE. The asymmetrical compound **B-13** was the product of **B-1** reacting with one equivalent 4F-TCNQ.

The charge transfer system of all of the symmetrical and asymmetrical compounds was changed. Because of this, different internal interactions were generated. Nuclear magnetic resonance, infrared, and mass spectrometry were used for the characterization of all of these compounds. This characterization confirmed their chemical structures.

Different photoelectric properties were represented by products of different conjugated structures as well as different electron-withdrawing groups having high non-linear performance.

### 3.2. UV-Vis Spectroscopy

**Figure 1:**
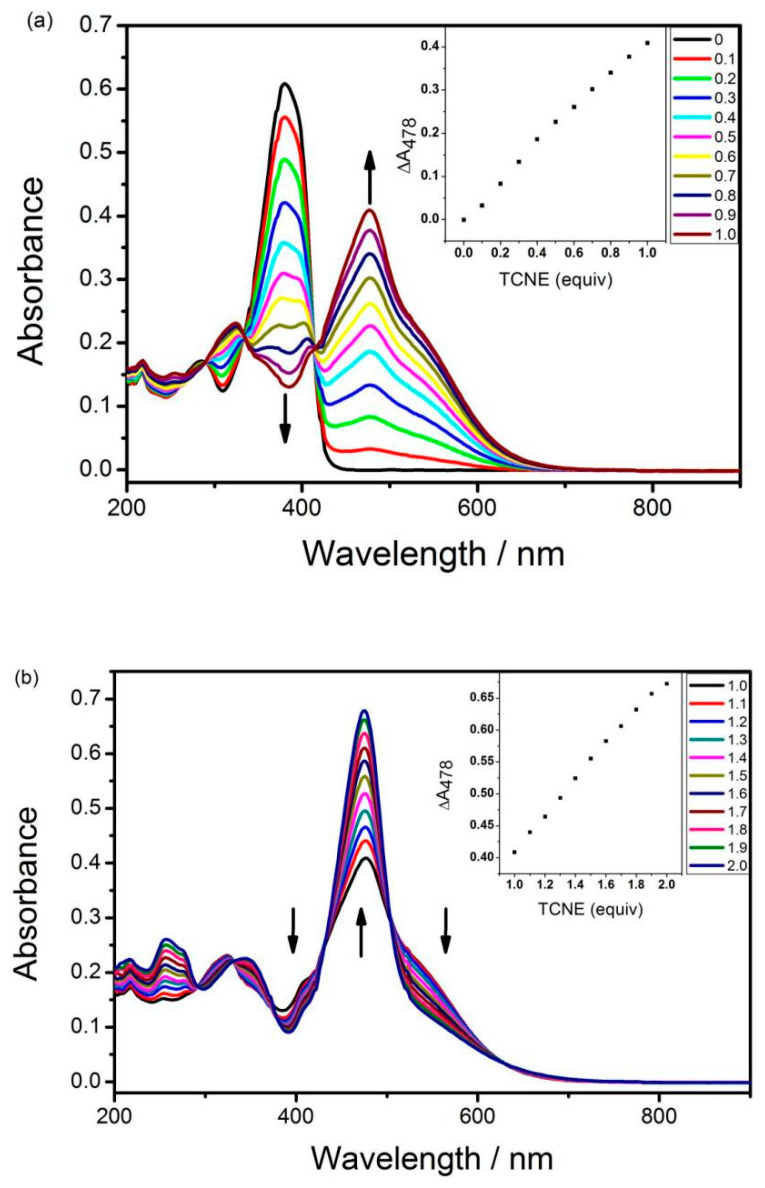
B reaction with quantitative TCNE in CH_2_Cl_2_, rt, to prepare **B-11**. (**a**) Equivalence ratio 0 to 1.0; (**b**) equivalence ratio 1.0 to 2.0; insertion depicts the change in absorbance by the addition of TCNE at 478 nm.

Ultraviolet/Visible spectroscopic titration of **B** (compound) was used to investigate click reaction. TCNE having strong electron-acceptor groups was added quantitatively in the CH_2_Cl_2_ solution of **B**. The change in both UV/Vis spectra and the equivalence ratio (0 to 1.0) is shown in Figure 1a. The absorption peak at 380 nm started to decrease when TCNE at a 0.1 equivalence ratio was added. At 478 nm, a new relatively weak peak was shown because of click product **B-1**. Upon increasing the amount of TCNE, a significant increase in the absorption peaks was recorded for **B-1**. However, absorption intensities for precursors decreased gradually. Figure 1b depicts the change in UV/Vis and the equivalence ratio (1.0 to 2.0). A linear increase in the absorption peak was recorded at 478 nm (Figure 1 Inset).

The presence of **B-11** (click product) narrowed the peak width gradually. The existence of isosbestic points at 334 nm and 414 nm is shown in Figure 1a, as well as at 436 nm and 502 nm in Figure 1b. It can be observed that no side reaction took place. Ultraviolet/Visible spectroscopic titration experiment proved that the synthesis of **B-11** was a selective two-step process. Firstly, one side of the two alkynyl groups of B reacted completely with click reagent TCNE, then the other side of the two alkynyl groups reacted to yield **B-11**. This proved the simplicity, high efficiency, and quick reaction of the click chemistry.

**Figure 2:**
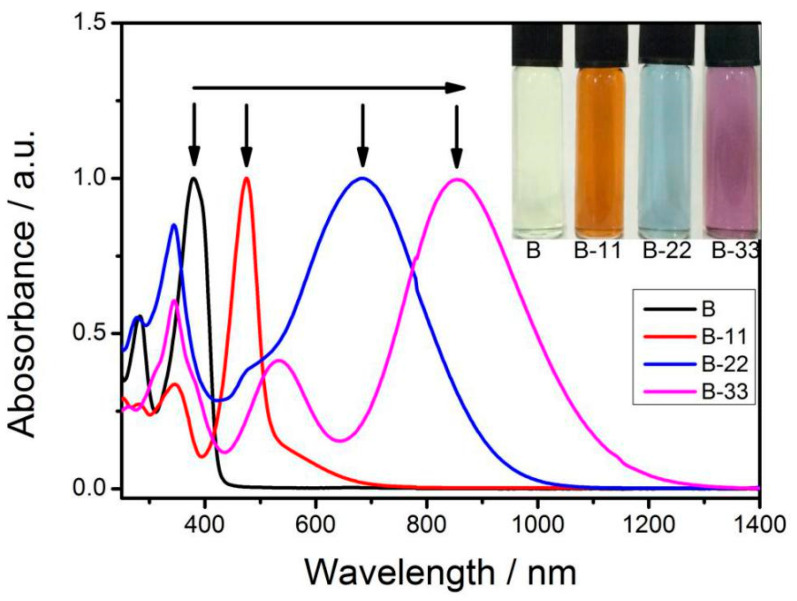
Ultraviolet/Visible captivating spectra of **B**, **B-11**, **B-22**, and **B-33** in CH_2_Cl_2_ solution, rt.

The solution color in CH_2_Cl_2,_ as well as Ultraviolet/Visible captivating spectra of **B**, **B-11**, **B-22**, and **B-33**, is presented in Figure 2. A yellow compound, **B** reacting with two equiv click reagents TCNE, TCNQ, and 4F-TCNQ, yielded brown **B-11**, light blue **B-22**, and purple **B-33**, respectively. The maximum absorption wavelengths and cut-off wavelengths of all of the compounds are shown in Table 1. The maximum absorption peaks of **B-11**, **B-22**, and **B-33** bathochromically shifted to 478 nm, 691 nm, and 867 nm, respectively, after the addition of click reagents.

This was due to the improvement in electron affinity by introducing substitutes (strong electron-withdrawing) and enhancement in conjugated lengths [31,32]. The maximum absorption peak of **B-33** exhibited a more significant redshift as compared with **B-11** and **B-22**. This is due to the addition of CN and F atoms having a stronger affinity for electrons into **B**, and the addition of longer conjugated lengths. Although four compounds have similar structures, their optical properties were found to be completely different. This showed that they have different NLO properties and effects of the conjugation system on NLO response.

**Figure 3:**
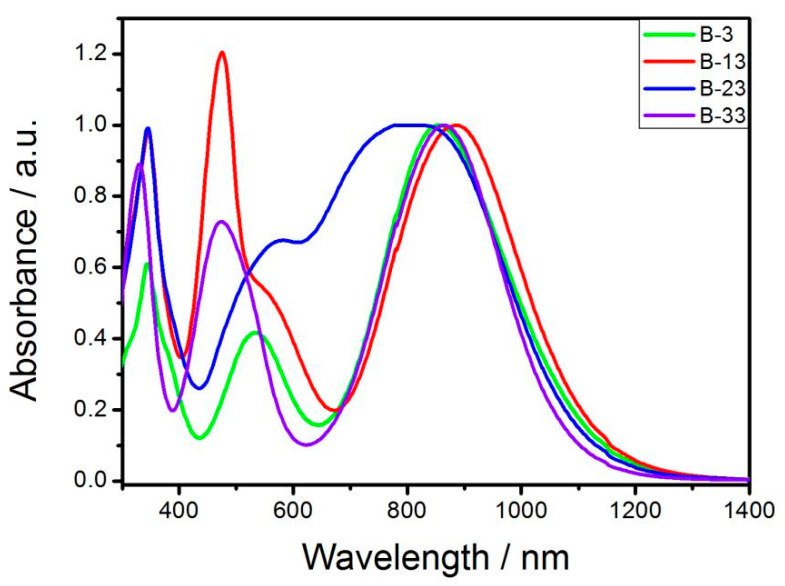
Ultraviolet/Visible absorption spectra of **B**, **B-11**, **B-13**, **B-23**, and **B-33** in CH_2_Cl_2_ solution, rt.

Figure 3 exhibited the absorption spectra of different products **B-3**, **B-13**, **B-23**, and **B-33**. All of the compounds showed an absorption peak at about 858 nm due to the introduction of the same functional group (4F-TCNQ). Compared with **B-3**, the absorption peak of **B-13** at about 888 nm underwent slightly bathochromic shifts, which was due to the small increase in the conjugation length by reacting with TCNE.

Compared with **B-3**, the absorption peak of **B-33** at about 888 nm underwent slightly blue shifts due to the electronic collaborations among symmetrical groups. In summary, both conjugation length and symmetry of the molecule affected its UV/Vis absorption.

### 3.3. Electrochemistry

**Figure 4:**
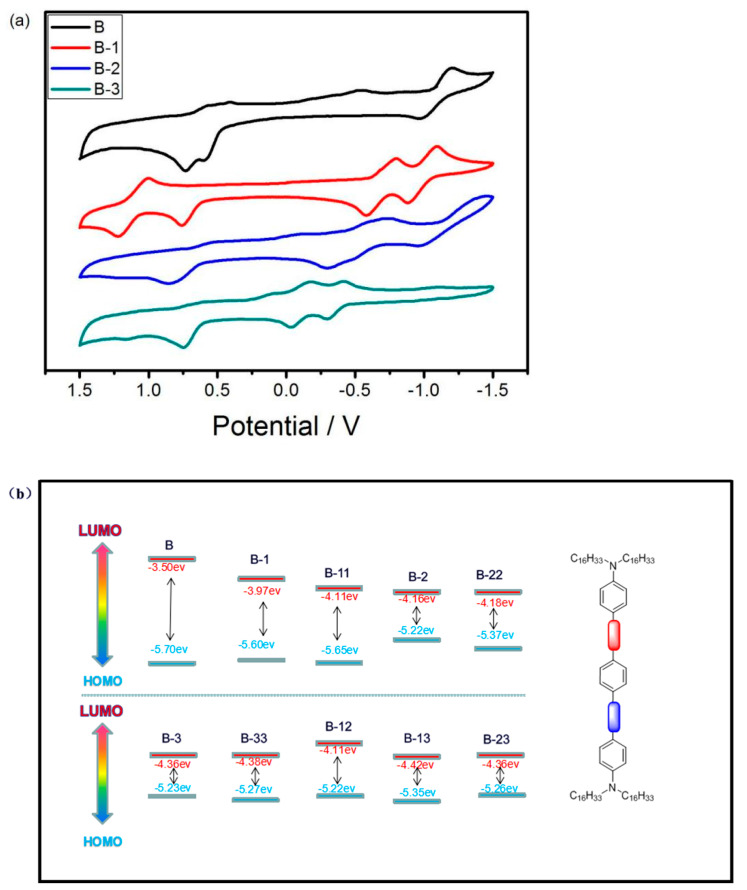
(**a**) Cycle voltammograms of B-X (**B**, **B-1**, **2**, and **3**) in CH_2_Cl_2_/Bu_4_NPF_6_ at rt; (**b**) LUMO and HOMO energy levels of all the compounds.

Among the key aspects that affect the third-nonlinear polarization ability of carbon-based compounds, electron delocalization is one of them. Because of the different LUMO/HOMO energy levels, electron delocalization showed a significant change [33,34,35]. Cyclic voltammetry (CV) was used to examine the electrochemical properties. The CV of **B** reacting with 1 equiv different click reagents (TCNE, TCNQ, and 4F-TCNQ) is shown in Figure 4a.

In the UV/Vis absorption spectroscopy (JASCO Corporation, Tokyo, Japan), the bathochromic shift from **B-1** to **B-3** in the charge-transfer band showed that click post functionalization might control the energy levels [36,37,38]. The optical and electrochemical properties of all products are shown in Table 1. The LUMO levels, as well as electrochemical band gaps, were computed via the inception of redox capacities.

Using cut-off fascination wavelengths of products, the optical band gaps were calculated. It was shown that the electrochemical band gaps of ten products were in the fair covenant by the ocular band gaps. The LUMO as well as HOMO levels of ten products are shown in Figure 4b.

It was revealed that the reactions of acetylene groups with click reagents lowered the compounds’ LUMO levels and band gaps. This was due to the robust electron-withdrawing skill and prolonged π-conjugation [39,40]. It was obvious from the stumpy HOMO levels and constricted energy gaps that strong electron transition was possessed by all of the tested compounds [41,42].

Comparison between **B-1**, **2**, and **3** with B showed that their LUMO levels and energy gaps were decreasing. Among all, **B-3** exhibited a lower LUMO level and narrower energy gap because of its larger π-conjugation and the presence of CN and F atoms (atoms having a strong affinity for electrons).

Comparing the products, **B-1**, **11**, **2**, **22**, **3**, and **33** showed that, when one or two acetylene groups reacted with the same click reagents, similar LUMO levels and band gaps were recorded. This indicated that the π-conjugation and electron-withdrawing ability of molecules had a greater influence on the electrochemical properties than the molecular symmetry.

### 3.4. Non-Linear Optics (NLOs)

The Z-scan practice was used for studying the non-linear optics responses. Non-linear refractive behavior and non-linear absorption of all the products were tested through the closed Z-scan and the open Z-scan measurements, respectively. Z-scan technology can simply and quickly test the NLO refraction or NLO absorption curve of materials, and the NLO absorption coefficient and NLO refraction coefficient of related materials can be effectively obtained through relevant calculation, so as to further obtain the third-order NLO coefficient of materials [43,44].

A 10^−6^ molar solution of CH_2_Cl_2_ was used for measuring all samples. Under these tentative environments, the solvent did not display any third-order nonlinearities. The reported equation presented in the supporting data was used to measure the NLO captivation as well as refraction. The data of Z-scan experiments are shown in Table 2.

**Figure 5:**
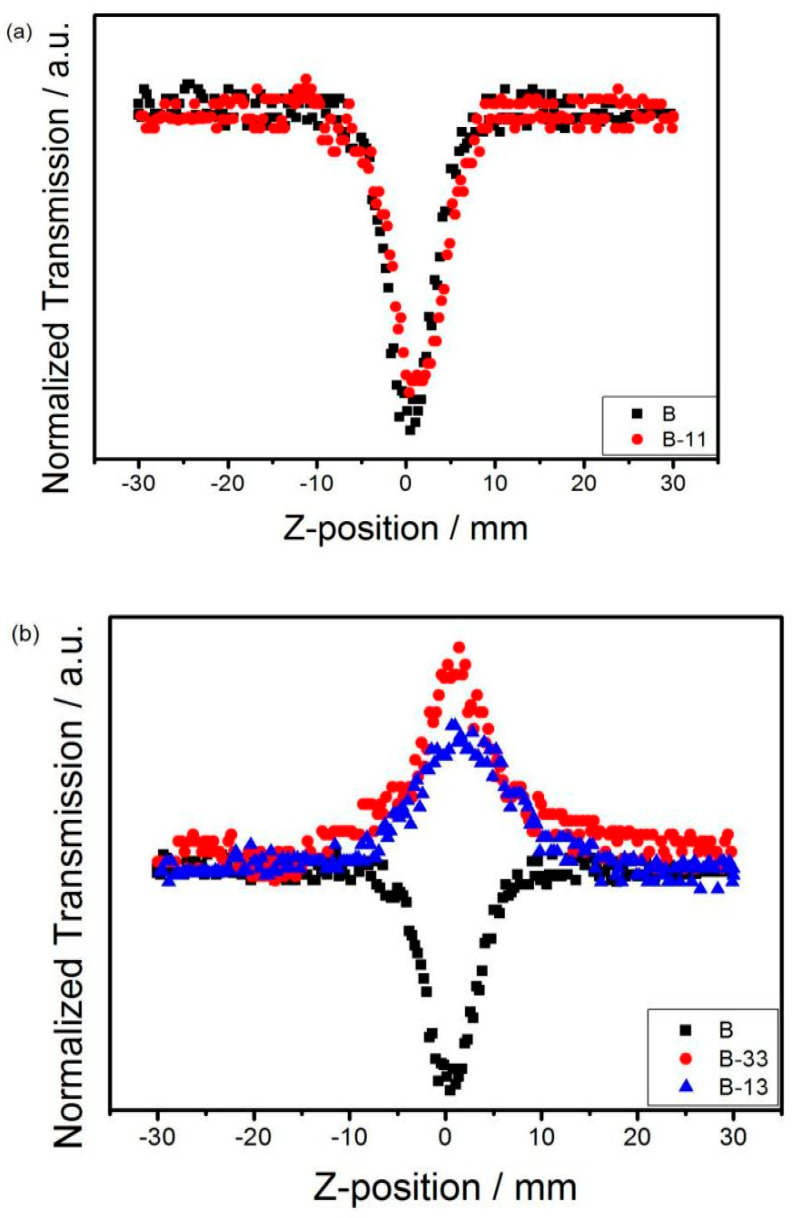
Regularized locked orifice Z-scan traces of the compounds (**a**) **B** and **B-11**; (**b**) **B** and **B-33**, **B-13**.

Figure 5 showed the “open aperture” Z-scan experiment along with normalized transmittance curves of the compositions (**B**, **B-11**, **B-13**, and **B-33**). As shown in Figure 5a, **B** and **B-11** exhibited similar reverse saturated absorption behaviors, while values of NLO coefficient (β) for **B-11** were slightly smaller than **B** owing to the introduction of CN and larger π-conjugation [45]. A significant increase in NLO vulnerability was recorded as a result of the transformation in charge transfer inside the molecule. The non-linear absorption of **B**, **B-13**, and **B-33** is shown in Figure 5b. Comparing **B-13** and **B-33** with **B**, these two products presented a change from the non-linear reverse saturable to saturable absorption. A plausible reason for this effect was credited to the expanded π-conjugation and the addition of robust electron-withdrawing groups F atoms and CN from 4F-TCNQ by the [2 + 2] click reaction.

The NLO coefficient (β) values of **B-13** and **B-33** were −0.9 × 10^−12^ and −1.2 × 10^−12^ m/W, respectively. Non-linear absorption coefficient values of **B-33** were larger than **B-13** because of the introduction of more fluorine atoms and larger π-conjugation.

The nonlinear absorption coefficient values of **B-33** were larger than **B-13** owing to the introduction of more fluorine atoms and larger π-conjugation.

**Figure 6:**
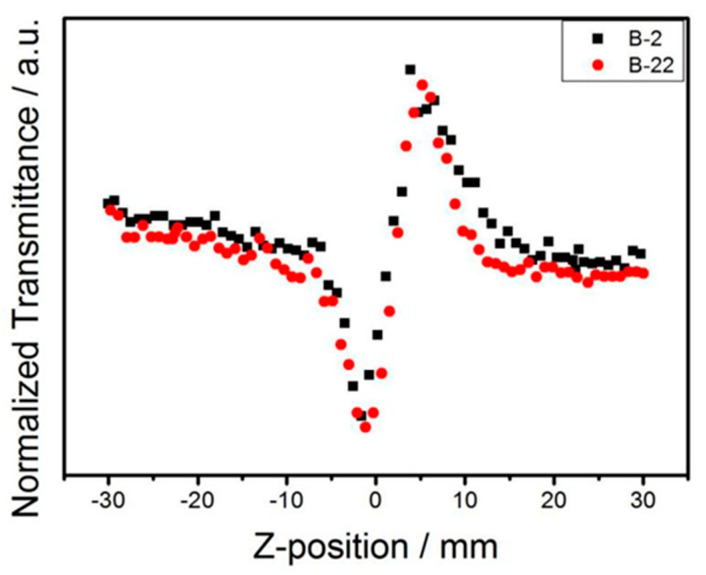
Regularized locked orifice Z-scan traces of the compounds.

The closed Z-scan curve of **B-2** and **B-22** is shown in Figure 6, which reflected their non-linear refractive behaviors. The two curves almost coincided and the non-linear refractive coefficient (n_2_) value of **B-2** and **B-22** was 2.5 × 10^−12^ m/W, showing that the molecule symmetry had an almost negligible consequence upon the non-linear refraction. The recent literature [15,16,22] showed the nonlinear absorption and refractive coefficient values of porphyrin derivatives and fullerene derivatives are in the order of the magnitude of 10^−12^ and 10^−19^ mW^−1^. Compared with these reported novel non-linear materials, the non-linear absorption and refractive coefficient values of our compounds reached the same magnitude.

## 4. Conclusions

We synthesized and purified ten different kinds of NLO materials by click chemistry. At the same time, NLO properties of the third order in both symmetrical, as well as asymmetrical compounds, were reported here. Compared with other organic nonlinear optical materials such as porphyrin derivatives and fullerene derivatives, their non-linear absorption and refractive coefficient values reach the same order of magnitude. Among them, the related benzene derivatives synthesized by different click reagents undergo a transition from saturable absorption to anti-saturable absorption of third-order nonlinear optical capability. The click reagents affect the arrangement and conjugated structure of electrons in the molecule, thereby further changing the third-order NLO properties of the material.

## Figures and Tables

**Table 1:** The electrochemical and optical properties of the compounds.

Materials	_λmax_^[a]^ (nm)	_λend_^[b]^ (nm)	E_g_^[c]^ (eV)	E_on_^ox [d]^ (V)	E_on_^red [d]^ (V)	E_g_^[e]^ (eV)
**B**	382	490	2.53	1.10	−1.10	2.20
**B-1**	478	734	1.69	1.00	−0.63	1.63
**B-11**	478	781	1.59	1.05	−0.49	1.54
**B-2**	698	1026	1.21	0.62	−0.44	1.06
**B-22**	691	1092	1.14	0.77	−0.42	1.19
**B-3**	867	1334	0.93	0.63	−0.24	0.87
**B-33**	867	1416	0.88	0.67	−0.22	0.89
**B-12**	475	1150	1.08	0.62	−0.49	1.11
**B-13**	475	1394	0.89	0.75	−0.18	0.93
**B-23**	823	1386	0.89	0.66	−0.24	0.90

[a]: wavelength for extreme fascination. [b]: wavelength for cut-off absorption. [c]: optical band breaks, (Ultraviolet/Visible captivating bands). [d]: onset of the redox peak (cyclic voltammograms). [e]: electrochemical band gaps.

**Table 2:** NLO properties of the compounds.

Sample	β	n_2_	Imχ^(3)^	Reχ^(3)^	χ^(3)^
	×10^−12^ m/W	×10^−19^ m/W	×10^−13^ esu	×10^−13^ esu	×10^−13^ esu
**B**	1.1	2	0.24	1.02	1.05
**B-1**	−4.5	-	−0.98	-	-
**B-11**	1	−3.6	0.22	−1.84	1.85
**B-2**	-	2.5	-	1.28	-
**B-22**	-	2.5	-	1.28	-
**B-3**	-	1.8	-	0.92	-
**B-33**	−1.2	-	−0.26	-	-
**B-12**	-	-	—	-	-
**B-13**	−0.9	2.3	−0.20	1.18	2.28
**B-23**	-	-	-	-	-

## Data Availability

The data presented in this study are available on request from the corresponding author.

## Sample Availability

Samples of the compounds **B-1**, **B-2**, **B-3**, **B-11**, **B-22**, **B-33**, **B-12**, **B-13** and **B-23** are available from the authors.
